# Soil Organic Carbon Redistribution by Water Erosion – The Role of CO_2_ Emissions for the Carbon Budget

**DOI:** 10.1371/journal.pone.0096299

**Published:** 2014-05-06

**Authors:** Xiang Wang, Erik L. H. Cammeraat, Paul Romeijn, Karsten Kalbitz

**Affiliations:** Earth Surface Science, Institute for Biodiversity and Ecosystem Dynamics, University of Amsterdam, Amsterdam, The Netherlands; DOE Pacific Northwest National Laboratory, United States of America

## Abstract

A better process understanding of how water erosion influences the redistribution of soil organic carbon (SOC) is sorely needed to unravel the role of soil erosion for the carbon (C) budget from local to global scales. The main objective of this study was to determine SOC redistribution and the complete C budget of a loess soil affected by water erosion. We measured fluxes of SOC, dissolved organic C (DOC) and CO_2_ in a pseudo-replicated rainfall-simulation experiment. We characterized different C fractions in soils and redistributed sediments using density fractionation and determined C enrichment ratios (CER) in the transported sediments. Erosion, transport and subsequent deposition resulted in significantly higher CER of the sediments exported ranging between 1.3 and 4.0. In the exported sediments, C contents (mg per g soil) of particulate organic C (POC, C not bound to soil minerals) and mineral-associated organic C (MOC) were both significantly higher than those of non-eroded soils indicating that water erosion resulted in losses of C-enriched material both in forms of POC and MOC. The averaged SOC fluxes as particles (4.7 g C m^−2^ yr^−1^) were 18 times larger than DOC fluxes. Cumulative emission of soil CO_2_ slightly decreased at the erosion zone while increased by 56% and 27% at the transport and depositional zone, respectively, in comparison to non-eroded soil. Overall, CO_2_ emission is the predominant form of C loss contributing to about 90.5% of total erosion-induced C losses in our 4-month experiment, which were equal to 18 g C m^−2^. Nevertheless, only 1.5% of the total redistributed C was mineralized to CO_2_ indicating a large stabilization after deposition. Our study also underlines the importance of C losses by particles and as DOC for understanding the effects of water erosion on the C balance at the interface of terrestrial and aquatic ecosystems.

## Introduction

Climate change will likely modify current precipitation regimes influencing the global carbon (C) cycle in relation to erosion processes [1], [2]. The length and intensity of droughts and the intensity of more sporadic rainfall events are predicted to increase for Western Europe [3], which will accelerate soil erosion. Soil erosion has significant impacts on the redistribution and transformation of soil organic carbon (SOC) within a landscape [4], [5]. Even now, there is no consensus whether soil erosion is acting as a net C sink [5], [6] or source [7] of atmospheric CO_2_. Therefore, quantitative assessments of soil organic C redistribution along geomorphic gradients and the processes involved become increasingly important in a changing climate to resolve this controversy [8]. It is crucial that such studies comprise the different processes associated with the redistribution of C along the slope including CO_2_ emissions as a result of changes in C mineralization upon erosion, transport and subsequent deposition. Based on such studies, complete C budgets of soils affected by erosion processes can be determined.

Soil erosion seems to preferentially remove fresh and more labile materials from C rich topsoils in upslope eroding positions, i.e. SOC with low density (e.g. free light fraction) and dissolved organic C (DOC) [7]–[10]. However, the fate of this organic C has rarely been studied. It is well known that most of the eroded sediments are re-deposited close to the source areas and in the catchment (e.g. [4], [11]). Deposition of C enriched sediments lead to accumulation of SOC in the downslope positions. The eroded and deposited C can be stabilized by interaction with minerals thereby decreasing mineralization of deposited C in soil profiles [12]. In addition, soil erosion could affect dissolved organic carbon (DOC) dynamics in soils. Wang et al. [12] found higher DOC concentration at eroding sites in comparison to depositional sites.

Soil erosion drastically influences not only lateral SOC distribution within a landscape but also vertical CO_2_ fluxes into the atmosphere [7], [10]. Van Oost et al. [5] summarized at least three key mechanisms controlling the net flux of C between the soil and atmosphere: 1) dynamic replacement of SOC at the eroding sites [6]; 2) deep burial of SOC rich topsoils at depositional sites [4], [13]; 3) enhanced decomposition of SOC because of the chemical or physical breakdown of soil during detachment and transport [7]. Particularly, the second and the third mechanisms should be susceptible to changes in the precipitation regime.

A key uncertainty of erosion-induced C loss is C mineralization resulting from the breakdown of soil aggregates as a direct response to extreme precipitation [7], [14], [15]. During a given erosion event, rainfall leads to breakdown of aggregates and releases the encapsulated C due to flow shear and raindrop impact [14]. Some studies suggest that aggregates breakdown by raindrop impact and wetting is mainly caused by initial fast slaking [16] or welding [17]. However, the extent of additional CO_2_ fluxes from breakdown of aggregates due to erosion is still largely unknown. Franzluebbers [18] estimated a 10–60% increase in CO_2_ evolution from various soils after breakdown of aggregates during 0–3 days. Polyakov and Lal [14] suggested that mainly the breakup of initial soil aggregates by erosive forces is responsible for increased CO_2_ emission. However, conducting a set of rainfall simulation experiments, Bremenfeld et al. [19] recently suggested that interill erosion and associated soil aggregates breakdown have no prominent effect on soil respiration *in situ*. Therefore, effects of erosion-induced breakdown of aggregates on CO_2_ evolution need to be further assessed.

Estimates of soil and SOC redistribution and associated CO_2_ emissions show a large spatial and temporal variability. As field SOC and CO_2_ fluxes of soils under erosion strongly depend on temporal variability of environmental conditions (e.g. location, soil management, initial soil moisture, and rainfall event characteristics) rainfall simulations under controlled laboratory conditions may help to shed light on C flux processes. Several rainfall simulation experiments have attempted to investigate soil erosion and associated SOC dynamics [20]–[24]. Jacinthe et al. [24] determined mineralization of SOC in runoff under no-till, chisel till and moldboard plow conditions with rainfall simulation approach. Van Hemelryck et al. [23] experimentally simulated three typical agriculture erosion events to quantify CO_2_ emission. So far, however, there is no direct process assessment on combining effects of erosion, transport and subsequent deposition on C redistribution including vertical CO_2_ fluxes. Changes in SOC pools indicative for important mechanisms of SOC redistribution and differing in their stability against microbial decay are not well known.

To get a better process understanding of soil erosion, transport and deposition on the redistribution and mineralization of SOC, the main objective of the present study was to determine SOC redistribution and a complete C budget of a loess soil affected by water erosion using a pseudo-replicated rainfall simulation experiment under standardized conditions. The following processes were studied and considered in our C budget:

- We determined SOC mineralization by measuring CO_2_ emissions at different slope positions.
- We analyzed soil and C redistribution along the slope including potential export into aquatic ecosystems. We measured C enrichment in the redistributed sediment. In order to test the hypothesis that POC is preferentially eroded and exported into aquatic ecosystem we fractionated SOC by density into particulate organic C (free POC, C not bound to minerals) and mineral associated organic C (MOC).
- Finally, we analyzed concentrations of DOC in soil solutions at different positions of the slope and in runoff and determined above and belowground lateral DOC fluxes.

## Materials and Methods

### Ethics Statement

The experimental station ‘Proefboerderij Wijnandsrade’ (The Netherlands) permitted access to their land and allowed for taking soil sample material from their cereal fields for the research carried out.

### Site Description and Sampling

The loess soil was collected from an agricultural field with winter wheat in South Limburg (50°53′58. 42″N, 5°53′16. 23″E), The Netherlands in May 2011. South Limburg is part of the European loess belt and has a temperate maritime climate. This region has a mean annual precipitation of 825–850 mm [25] and a mean annual temperature of 10.2°C. The sampled soil has a silty loam texture, and is classified as a Haplic Luvisol [26]. In the present study, the top 10 cm of the *Ap* horizon was collected and sieved over an 8 mm mesh to homogenize the soils and to keep aggregates intact as much as possible. Agricultural management at the sampled site is characterized by a potato-winter wheat-beet-winter wheat rotation. Soils are plowed 30 cm by a cultivator in spring and conventional tillage was applied in winter (including 30 cm plowing). The basic physical and chemical properties of the used soil are shown in Table 1.

**Table 1:** Basic properties of the loess soil used in the experiment. Results are shown as mean and standard error of three replicates.

Depth (cm)	Bulk density (g cm^−3^)	pH	SOC^a^ (%)	TN^b^ (%)	C/N	Soil texture (%)		
						Sand	Silt	Clay
0–10	1.28 (0.05)	6.5 (0.06)	1.07 (0.06)	0.11 (0.01)	10.3 (0.7)	8.6	82.2	9.2

a : Soil organic carbon.

b : Total nitrogen.

### Soil Analysis

Field bulk density was estimated from undisturbed 100 cm^−3^ cores that were oven-dried at 105°C for 24 hours [27]. Grain size distribution of soils was obtained using a particle size analyser (Micromeritics, SediGraph 5100, Norcross, USA). Soil pH (1∶2.5 in H_2_O) was measured with a multi-parameter analyser (CONSORT C832, Abcoude, The Netherlands). Soil water content was continually determined by a multi-channel Metallic TDR cable tester system [28]. Carbon and nitrogen (N) contents in bulk soils, sediments and density fractions were determined using a C and N analyser (Elementar VarioEL, Hanau, Germany).

### Experimental Design

**Figure 1:**
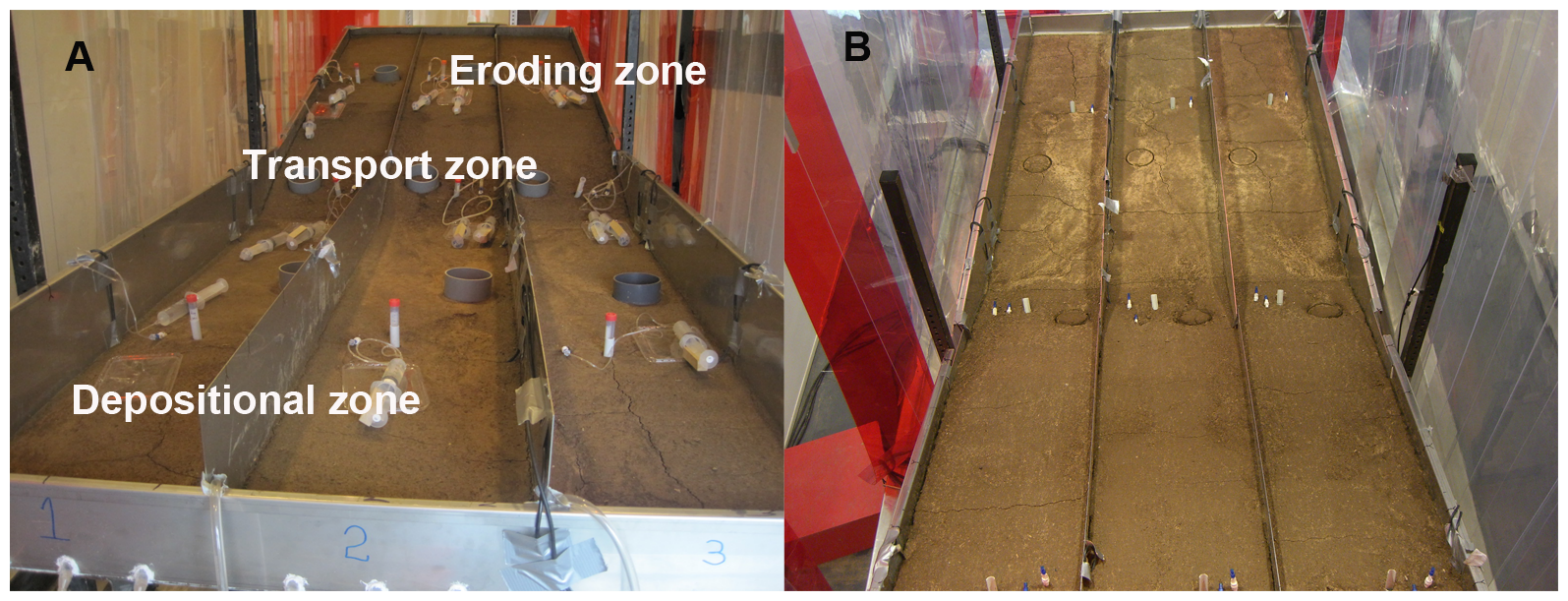
Photographs of the experimental setup and sampling locations along the experimental flume. It included the eroding, transport and depositional zones of the flume. A shows the lateral view; B shows the vertical view.

The erosion experiment was carried out using a 1.25 m×3.75 m experimental stainless steel flume (Figure 1). The upper 1.75 m had a slope of 15° (upslope position) and the lower 2 m had a slope of 2° (downslope position). To assess the effects of erosion, transport and subsequent deposition on redistribution of soils and C along the erosion slope, the experimental flume was divided into three zones according to the positions of the slope and observed results of sediments redistribution (Figure 1): 1) the eroding zone, at the upper half of the upslope position; 2) the transport zone, at the lower half of the upslope position and the upper half of the downslope position; 3) the depositional zone, at the lower half of the downslope position of the flume. We used a static definition of the different zones as dynamic measurement locations would have disrupted the soil surface. We recognize that these zones can change during the event and between events and that during events in every zone also local deposition and re-entrainment will occur.

The entire flume was subdivided into three parallel replicates of 40±2 cm wide. The soil was laid on top of a 2 cm thick layer of inert quartz sand to allow water to drain away. On top there was a 20 cm layer of soil on the upper (erosion) section where soil was supposed to erode and a thinner (5 cm) soil layer on the lower deposition section to allow for material deposition. On the transport section there was a gradual transition from 20 to 5 cm soil layer. While placing the air-dried soil it was compacted for every 2 cm, using a hammer and wooden piece of board (30×30 cm) to distribute the applied force. The compaction was such that it approached bulk density under field conditions (1.28 g cm^−3^). In addition, there were three controls. Three control buckets (diameter 34 cm) were filled with a 20 cm loess soil layer on top of a 2 cm quartz sand layer, similar to the main flume. These control buckets were also compacted to the same bulk density. The buckets were placed next to the flume so that they received the same rainfall as well, but no lateral displacement of soil material took place.

The soil layer was pre-wetted to an initial standard moisture contents (Table 2) in 10–15 min to initiate runoff generation prior to commencing the real rainfall experiment. Four 18-minutes rainfall events were carried out at a monthly time interval. Measurements were carried out every 2 minutes during rainfall simulation. Rainfall was simulated with two nozzles (Lechler 460 788) applying at 1600 hPa demineralized water using an average rainfall intensity of 41.8±1.9 mm h^−1^. A rainfall event with this intensity and duration of 18 minutes has a return period of about 2 years [29]. Mean drop size of the applied rainfall was 2.0 mm (D_50_ = 2.0). With an average falling height of 1.8 m, the kinetic energy applied on the soil surface was 12.5 J m^−2^ mm^−1^. Demineralized water was used instead of tap water to prevent flocculation problems with dispersible soil material [30], [31]. As the total load of ions in rainwater is very low (the annual average electrical conductivity EC_25_ was below 20 µS cm^−1^ at the official Dutch sampling site Beek [32], about 10 km from the soil sampling site) the physico-chemical impact of demineralized water on soil particles is considered to be the same as for rain water. The temperature was kept as constant as possible (18.1±0.9°C).

**Table 2:** Initial soil water contents (m^3^/m^3^) before and after pre-wetting before starting the rainfall simulation.

Zones	Event 1		Event 2	Event 3	Event 4
	Before	After	Before	After	Before	After	Before	After
Eroding	0.25	0.32	0.27	0.33	0.26	0.31	0.26	0.31
Transport	0.39	0.44	0.36	0.42	0.33	0.39	0.30	0.37
Depositional	0.36	0.48	0.33	0.46	0.30	0.42	0.23	0.44

### Sampling during Erosion Experiments

Sediment traps were installed in the middle of the eroding, transport and depositional zones respectively with entrance of the traps at the upslope side and at the same level as the soil surface to capture mobilized sediment in overland flow (Figure 1). The sediment traps were modified 12 ml Polypropylene screw cap tubes (Greiner Bio-One GmbH, Frickenhausen, Germany). The traps had a small diameter to minimize disturbance to overland flow and resulting erosion patterns. An opening in the side was made to collect mobilized sediments. The sediment traps were sampled every two minutes and the collected materials were transferred to containers, oven-dried at 35°C, weighed and later analysed for C and N contents.

Runoff and sediments were collected from weirs at the end of the flumes at 2-min intervals once continuous runoff had developed. Total runoff was collected using a polystyrene gutter that was installed at the lowest part of the experimental flume. The contents of the flume were then pumped into V-notched bottles to measure flow rates using a simple siphon pump made of Tygon R-3603 tubes (Saint-Gobin, Courbevoie, France). The lower end was constrained to 4 mm diameter to provide a constant flow velocity, without risking clogging by larger soil particles and keeping effects on the aggregation of the sediments limited. The V-notched bottles overflowed into sampling boxes which were replaced every two minutes or when the sampling box was full.

At the lowest end of the flume, three holes per replicate flume were present at the level of the sand drainage layer to collect through flow. Through flow was defined as the lateral underground flow in contrast to the overland flow. The holes were covered from the inside by a 63-µm stainless steel mesh allowing water to pass through, but to prevent clogging up. On the outside of the walls attached tubes drained into bottles, similar to the runoff setup.

### Sampling after Erosion Experiments

#### Density Fractionation of Bulk Soils and Exported Sediments

After four rainfall events the 0–2 mm topsoils at the eroding, transport and depositional zones and sediments exported during the first and fourth events were fractionated into three fractions by a sodium polytungstate (NaPT) solution with a density of 1.6 g cm^−3^: the free light fraction (fLF) which consisted of large, undecomposed or partly decomposed root and plant fragments, the light fraction occluded in aggregates (oLF) and the heavy fraction (HF), which was associated with minerals [33], [34]. Soil organic C in fLF, oLF and HF are defined as fPOC, oPOC and MOC, respectively. Particulate organic C (POC) is the C not bound to soil minerals including both fPOC and oPOC. The oPOC represents C sequestered in aggregates. Methods and procedures were followed as described in Cerli et al. [34]. All fractions were freeze-dried, homogenized and later analysed for C and N contents. Density fractionation was done in triplicate.

### Dissolved Organic Carbon (DOC)

To investigate dynamics of dissolved organic C in different soil depths and positions as affected by soil redistribution, soil moisture samplers (MACRO RHIZON 19.21.35, 9 cm porous, 4.5 mm OD, 0.2 µm, Wageningen, The Netherlands) were inserted in the eroding, transport and depositional zones of the flume. Each sampler was connected to a syringe (50 mL) to collect the soil solution. At the eroding and transport zones of the flume, soil solutions were collected at 4 cm and 9 cm depths. At the depositional zones soil solutions were sampled at 4 cm only because of the thinner soil layer on the lower deposition section. Soil solutions were sampled twice per week during the first week immediately after one rainfall event because of higher soil water moisture. As the soil dried, soil solutions were collected once per week. Concentrations of DOC were determined by a TOC analyser (TOC-V CPH, Shimadzu, Kyoto, Japan).

### Soil CO_2_ Efflux Measurements

Soil respiration was measured using a Portable Gas Exchange and Fluorescence System (LI- 6400XT; LICOR Biosciences, Lincoln, NE USA). In order to enhance the comparability of data, most CO_2_ efflux measurements were conducted in the afternoon between 17:00 and 19:00 at local time in PVC collars (10.2 cm in diameter and 7 cm in height). Soil CO_2_ efflux was determined before and after each rainfall simulation event. As the 7 cm high collars, necessary for the CO_2_ efflux measurements, would strongly affect the overland flow and erosion patterns during the rainfall event, the 7 cm high collars were replaced by smaller collars (same diameter but 1.5 cm tall). These were inserted at exactly the same place, to temporary fill the imprint of the high collar in the soil surface. The top of the collar was placed exactly equal to the soil surface, to minimize the disturbance of the sampling location by the CO_2_ measurements but still enabling to measure the CO_2_ efflux exactly at the same position later on. Overland flow was possible and erosion, transport and deposition processes at the surface of the area used for measuring CO_2_ were hardly affected by this strategy. Two to three measurements per site (i.e. per collar) were carried out each time. The number of replicated measurements per collar depended on the variation after the first two analysis with an additional measurement if the relative deviation of the second one was larger than 10%. Additionally, pre-experiments were carried out using the same loess soil to test impacts of soil depth on soil CO_2_ efflux. In these experiments the CO_2_ efflux was measured in columns with increasing soil thickness under constant soil moisture and temperature conditions. Results showed that soil depth did not have significant effect on soil respiration per soil weight up to a depth of 30 cm (data not published). Based on these results, all data measured in different experimental zones, and control soils, having different soil depths, were corrected to 20 cm soil layers in order to directly compare effects of erosion, transport and deposition on CO_2_ effluxes.

### Erosion-induced Carbon Budget

Fluxes of SOC and DOC were calculated by multiplying concentrations of SOC and DOC with the volume of the overland flow. Other parameters were calculated as follows: 
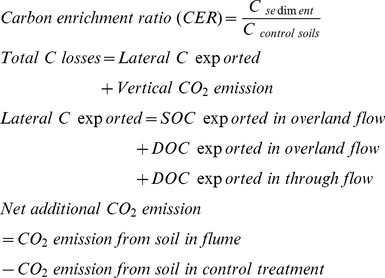



Based on the 4-month data we calculated annual C fluxes by linear extrapolation making comparisons with the literature easier. However, the shortcomings of such budgets based on short-term laboratory experiments only are obvious.

The definition of C source and sink areas for calculating the C budget was based on two experimental observations. After the fourth rainfall event, soil layers with relocated materials were clearly visible in the flume, particularly in the downslope part of the depositional zone (Figure 1B). In addition, we found that SOC was significantly depleted in the transport zone comparing with controls soils (cf. section results). Based on these two observations, the eroding and transport zones were defined as the C source area and the depositional zone and the runoff leaving the flume (exported into aquatic system) were defined as the C sink area. We calculated an erosion-induced SOC budget for the four rainfall events over the entire period using a mass balance approach (i.e. source  =  sink area). Changes in C distribution between the density fractions were appropriately considered by using the data of the original soil for the source area. This approach enabled us to include any changes in C redistribution between density fractions induced by erosion.

### Statistical Analyses

Differences in C enrichment ratios, amounts of sediment exported and DOC concentrations in overland flow were tested with one-way ANOVA and the Post-hoc Duncan test to differentiate between individual differences. The difference of CO_2_ effluxes measured in the 4-month period at eroding, transport and depositional zones of the gutter was tested by repeated measurement ANOVA. Averaged CO_2_ efflux in different experimental zones was compared using a one-way ANOVA. For all tests, a significance level of *P* = 0.05 was set using the Post-hoc Duncan test, unless otherwise indicated. The relationship between cumulative CO_2_ emission and DOC concentration was tested by two-tailed Pearson test. All statistical tests were performed using SAS software (Version 8.1) and SPSS (IBM Statistics 20).

## Results

### Loss of Sediment and Carbon Enrichment Ratios in Overland Flow

**Figure 2:**
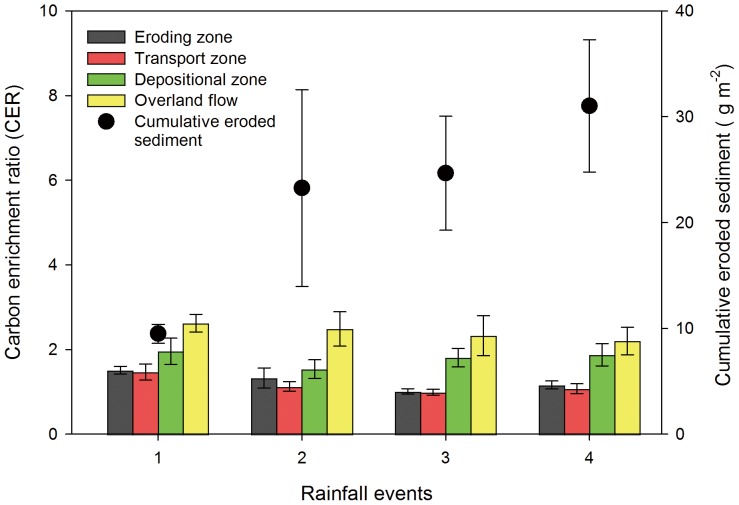
Average total eroded sediment per rainfall event exported by overland flow and carbon enrichment ratios (CER) during four rainfall events.

Total sediment losses in the overland flow increased during the course of the experiment from 9.5 g m^−2^ in the first event to 31.0 g m^−2^ in the fourth event (Figure 2). During the first rainfall event the average sediment concentration was 1.1±0.2 g L^−1^ and doubled to 2.3±0.8 g L^−1^ in the fourth event.

**Figure 3:**
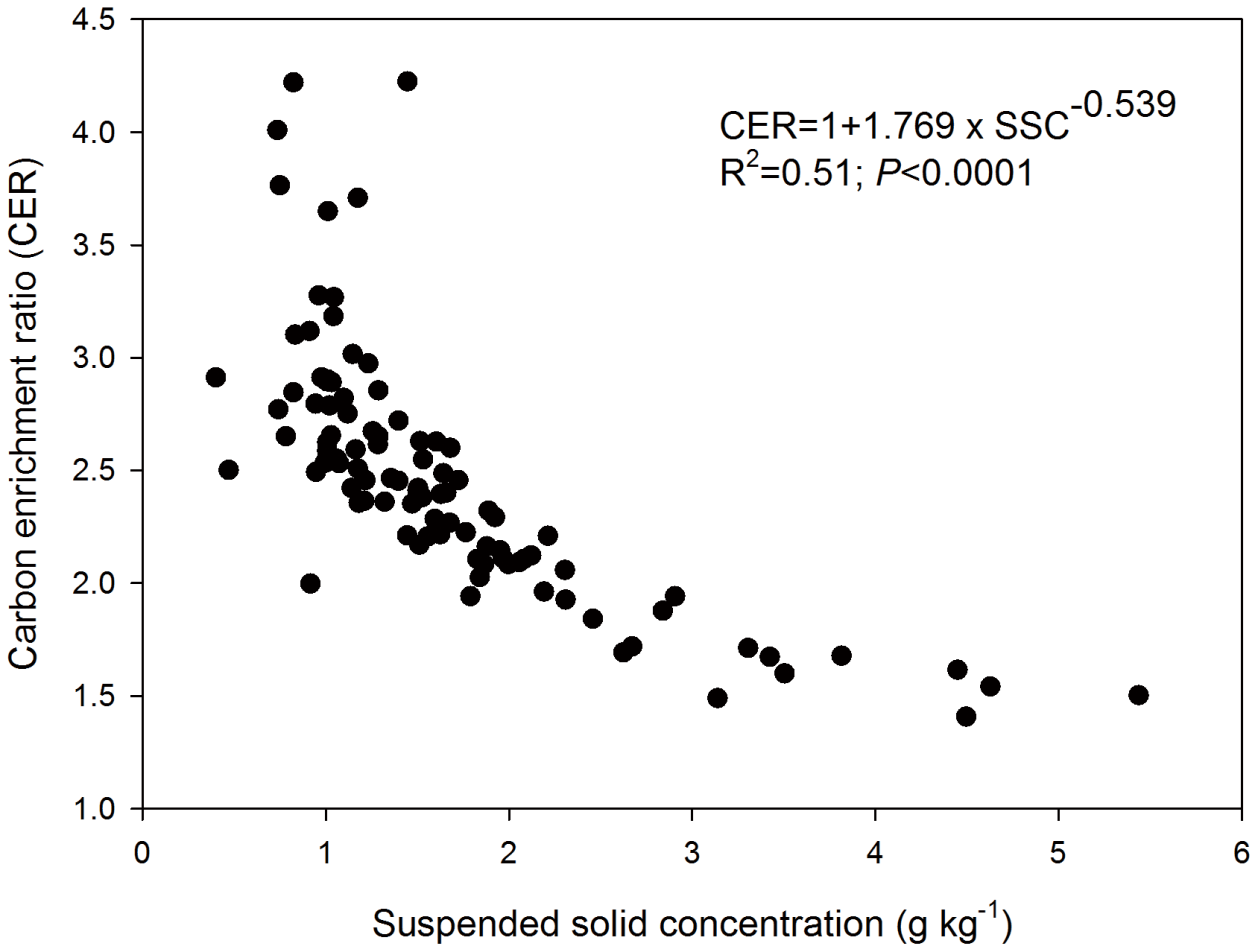
Relationship between carbon enrichment ratio (CER) and suspended solid concentration (SSC) in the overland flow.

Carbon enrichment ratios (CER) of sediment loads of overland flow trapped at the eroding, transport and depositional zones of the flume ranged from 0.8 to 2.9. The CER was significantly higher at the depositional zone compared to those of the eroding and transport zones (Table 3). Carbon enrichment was even stronger in the sediments of the runoff with CER between 1.3 and 4.0. Carbon enrichment ratios decreased with increasing concentrations of suspended solids in the overland flow (Figure 3). Concentrations of suspended solids were smaller at the beginning of each rainfall event, resulting in larger C enrichment but also in larger variation of the data.

**Table 3:** Carbon concentrations and specific carbon fractions of soils and sediments for different zones and events.

Zones	C concentration^a^	C concentration^b^			C enrichment ratio (CER)^c^				Relative proportion of MOC^d^
	(mg g^−1^ soil)	(mg C g^−1^ specific density fraction)			(-)				(% SOC)
	Bulk soils	fPOC	oPOC	MOC	Bulk soils	fPOC	oPOC	MOC	MOC
Control	10.0 (0.5)	134.3 (28.9)	162.3 (24.4)	8.0 (0.1)					91
Eroding	9.4 (0.2)	189.8 (26.3)	175.3 (20.4)	8.0 (0.4)	0.94	1.1	0.8	0.9	91
Transport	9.7 (0.2)	220.3 (60.3)	143.1 (31.5)	7.7 (0.2)	0.97	1.9	1.0	0.9	87
Depositional	10.4 (0.5)	205.0 (61.8)	175.5 (25.1)	7.9 (0.3)	1.04	1.6	0.9	1.0	90
Overland flow 1	22.9 (0.9)	151.2 (42.2)	345.5 (20.1)	17.3 (0.6)	2.30	3.9	3.2	2.2	86
Overland flow 4	16.6 (1.6)	219.1 (54.3)	296.4 (39.3)	13.9 (0.9)	1.67	2.2	2.3	1.6	88

C in free light fraction  =  free particulate organic C, fPOC; C in occluded light fraction  =  occluded particulate organic C, oPOC; C in heavy fraction  =  mineral-associated organic C, MOC. Results are shown as mean and standard error of three replicates.

a . Carbon concentration of bulk soils (mg C g^−1^ soil).

b . Carbon concentration of the three density fractions fPOC, oPOC and MOC in relation to the total weight of that specific soil fraction (mineral + C parts) (mg C g^−1^ soil fraction).

c . Carbon enrichment ratios, calculated on the basis of mg C soil fraction g^−1^ soil organic C.

d . Relative proportion of MOC (%SOC) in bulk soils, density fractions and sediments of overland flow for the first (Overland flow 1) and fourth rainfall event (Overland flow 4).

### Preferential Erosion and Deposition of Organic Carbon at the Soil Surface

After four rainfall events, a thin sedimentation layer was present in the depositional zone (approximately 2 mm thick) without any layering. However the depositional zone clearly showed patterns of deposition of finer grained materials along the flow lines of overland flow and the whole lower part of the gutter. Soil organic C concentration (mg^−1^ g soil) of the surface soil (2 mm) decreased by 6.0% at the eroding zone and increased by 3.9% at the depositional zone if compared to control soils (Table 3). Nevertheless, soil organic C concentration did not differ significantly between the control, the eroding, transport and depositional zones of the gutter. Also the relative distribution of C in density fractions of the soil was not affected by soil erosion. Most of the C (86% to 91%) was found in the heavy fraction, i.e. mineral associated organic C (MOC; Table 3). The rest was almost equally distributed between the free light fraction (particulate organic C in free light fraction  =  fPOC) and the fraction occluded within aggregates (oPOC). The free light fraction was significantly enriched in C at the transport and the depositional zone whereas the occluded light fraction (oLF) was depleted in C at the eroding zone (Table 3). The heavy fractions of surface soils in the flume did not significantly change in C contents.

In the sediments, all fractions were strongly enriched in C with the largest enrichment in the free light fraction. This C enrichment was smaller in the occluded and smallest in the heavy fraction and also decreased from the first to the last event (Table 3). However, the C content of the heavy fraction of the sediments (first event) was more than double the C content of the heavy fraction of the control soil (Table 3).

### Soil CO_2_ Efflux

**Figure 4:**
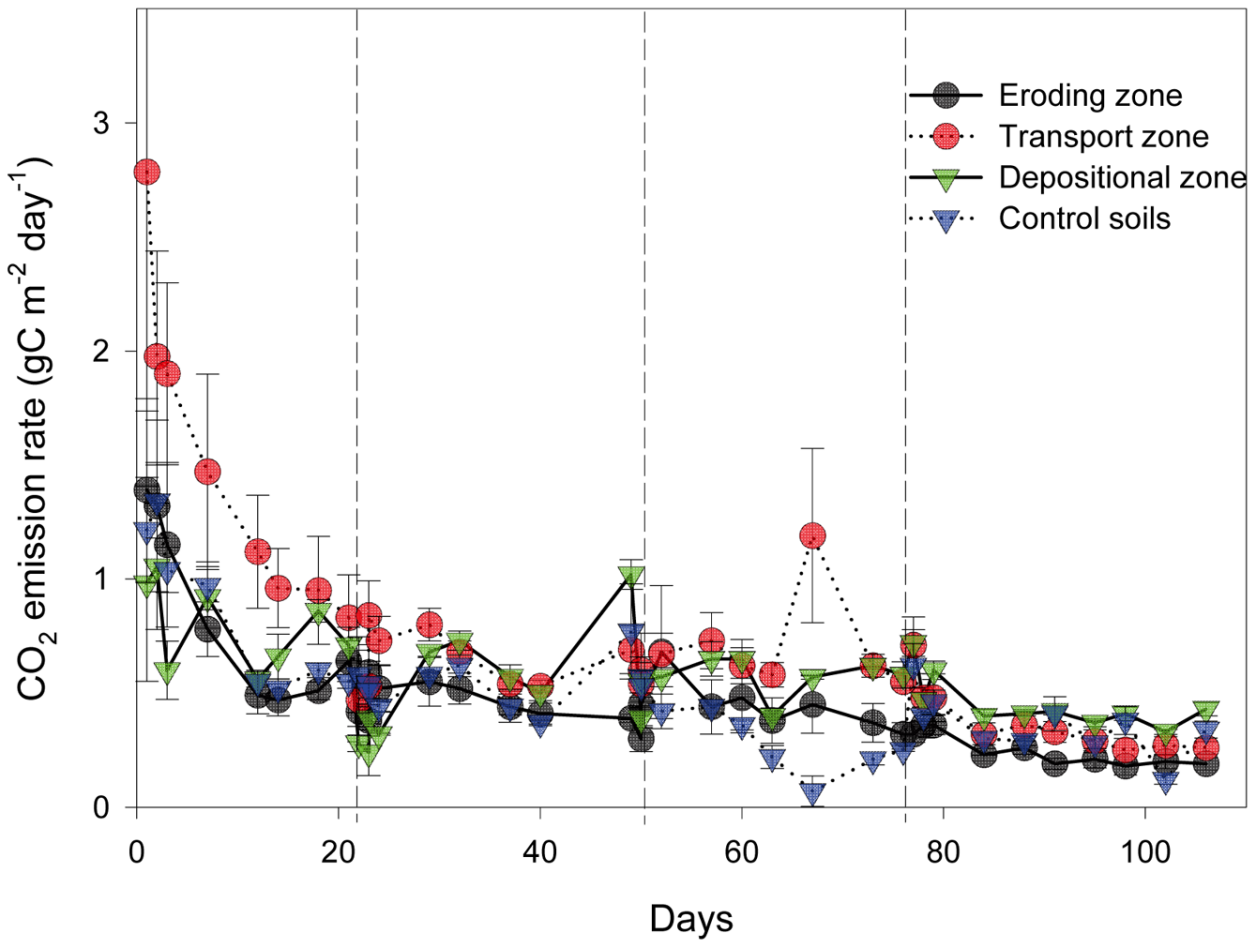
CO_2_ efflux at different zones of the gutter and control soil during four rainfall events. Solid line + circle represents the eroding zone; dotted line + circle represents the transport zone; solid line + triangle represents the depositional zones; and dotted line + triangle represents control soils. Values are mean± standard error of three replicates.

All measured CO_2_ efflux rates for the whole experiment ranged from 0.12 to 4.34 g C m^−2^ day^−1^ (Figure 4). During the entire experimental period, rates of CO_2_ emissions exhibited a similar behaviour in the eroding, transport and depositional zones and the non-eroded control with a sharp initial increase immediately after each rainfall event, followed by continuously decreasing rates thereafter. Rates of CO_2_ efflux significantly decreased with time during the four events (*P* = 0.001). The spatial and temporal variability of CO_2_ efflux rates was larger in the first rainfall event than during the other events.

**Figure 5:**
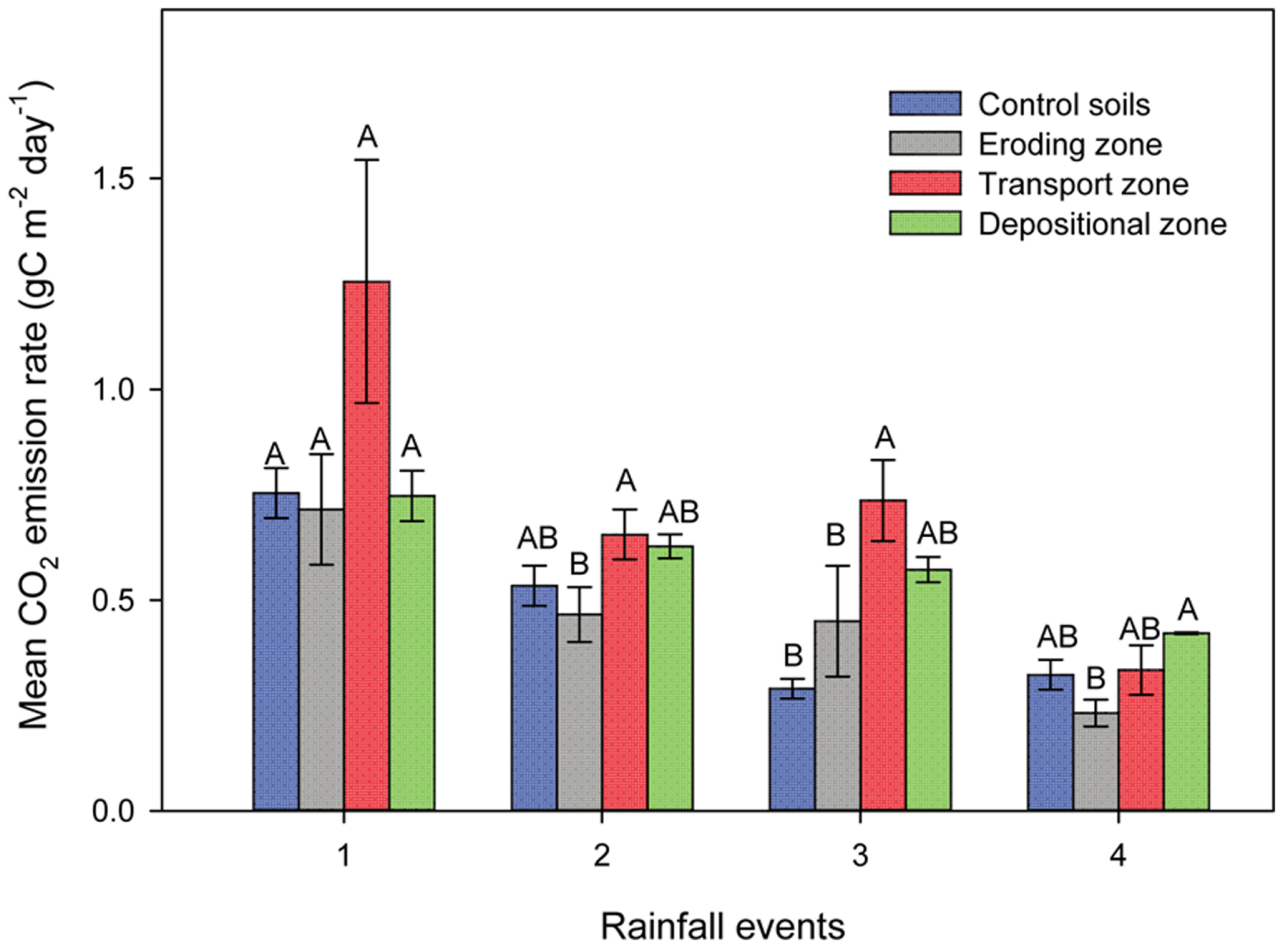
Mean cumulative CO_2_ emission at the eroding, transport and depositional zones and control soil. Different capital letters mean significant difference at a single rainfall event between the different zones. Values are mean± standard error of three replicates.

The largest mean CO_2_ efflux was observed in the transport zone during the first three rainfall events (Figure 5). In the fourth event, however, the depositional zone had the largest mean CO_2_ efflux. The relative differences of the mean CO_2_ efflux between the depositional and the eroding zones increased during the course of the whole experiment and became significant in the fourth event.

Cumulative CO_2_ fluxes in the eroding, transport and depositional zones ranged from 80 to 180, 116 to 317, and 146 to 204 g C m^−2^ yr^−1^, respectively. The largest mean cumulative CO_2_ fluxes (221 g C m^−2^ yr^−1^) were observed in the transport zone. Mean cumulative CO_2_ fluxes in the depositional zone (181 g C m^−2^ yr^−1^) were significantly larger than those in the control soils (*P* = 0.02) while CO_2_ fluxes in the eroding zone were similar in comparison to the control. The total losses of C as CO_2_ emission during the entire experiment accumulated to 1.8 to 2.9% of total soil organic C stocks.

### Dissolved Organic Carbon (DOC)

**Figure 6:**
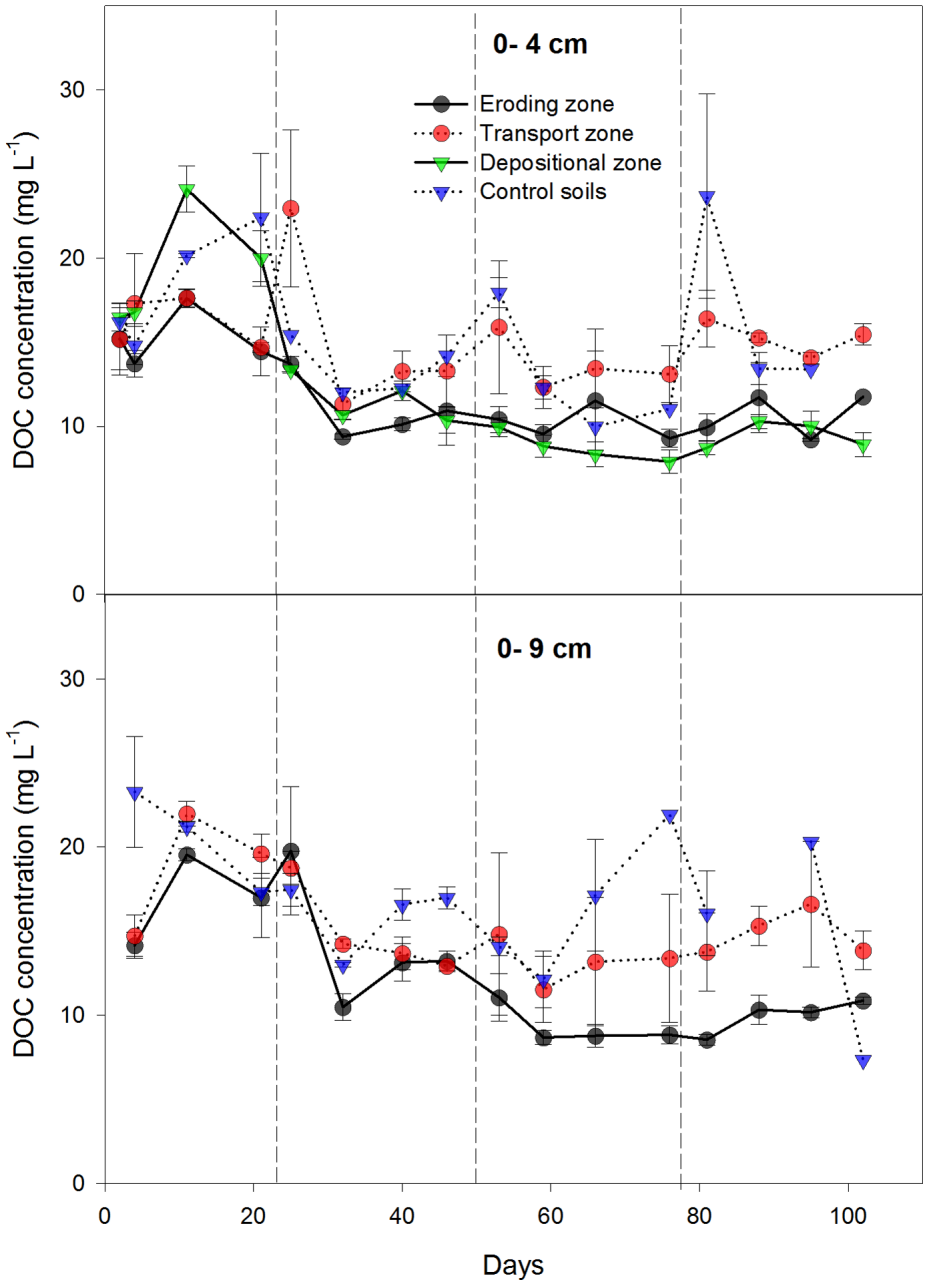
Dissolved organic carbon (DOC) concentrations. DOC solutions were collected at 0–4 cm and 0–9 cm depths of the eroding, transport and depositional zones of the flume during four rainfall events. Solid line + circle represents the eroding zone; dotted line + circle represents the transport zone; solid line + triangle represents the depositional zones; and dotted line + triangle represents control soils. Values are mean± standard error.

Concentrations of DOC in soil solutions at eroding, transport and depositional zones ranged from 7.1 to 25.9 mg L^−1^ during four rainfall events (Figure 6). In the shallow soil (4 cm depth), mean concentration of DOC decreased in the following order: transport zone (15.1 mg L^−1^) > control soils (14.3 mg L^−1^) > depositional zone (12.3 mg L^−1^) > eroding zone (11.8 mg L^−1^). However, only DOC concentrations in the depositional and eroding zones were significantly lower than those in the transport zone and the control. Mean concentrations of DOC in the deeper soil (10 cm) were almost equal as in the shallow soil and decreased in the following order (not statistically significant): control soils (16.8 mg L^−1^) > transport zone (15.2 mg L^−1^) > eroding zones (12.3 mg L^−1^). Concentrations of DOC in soil solutions of both depths showed distinct temporal patterns in all zones of the gutter. They increased at the beginning of each rainfall event, then decreased and increased again with time. This trend was less obvious during the first rainfall.

**Figure 7:**
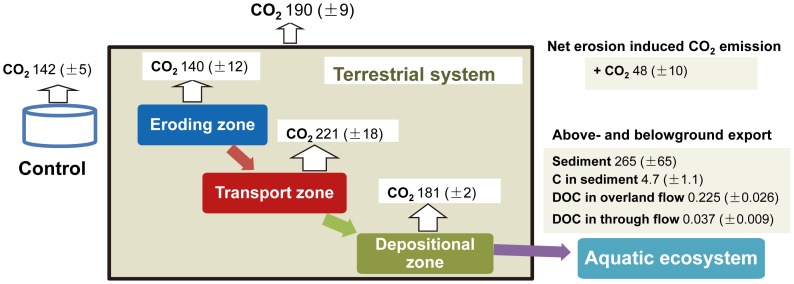
Conceptual diagram illustrating the total carbon budget as affected by soil erosion, transport and deposition in the four months rainfall simulation experiment. Fluxes were calculated on an annual base (interpolated from the 4-months experiments). The values were expressed as mean values and standard error of three replicates.

Concentration of DOC in overland flow remained constant during each single event, ranging from 0.3 to 8.3 mg L^−1^ and significantly decreased from the first to the third rainfall event (means of the four rainfall events: 7.2±0.4, 2.6±0.4, 0.9±0.7, 0.7±0.4 mg L^−1^, no further data shown). Cumulated DOC fluxes transported by overland flow were on average 0.23 g C m^−2^ yr^−1^ (Figure 7). The amount of C exported as DOC by overland flow was small, accounting for 0.014% of the total SOC stocks in the flume. Fluxes of DOC in through flow (i.e. 0.002% of total SOC stocks) were significantly smaller than overland flow.

## Discussion

### Preferential Transport and Deposition of Organic Carbon

As expected from the literature [8], [12], the soil of the eroding zone was depleted in C whereas the soil of the depositional zone and the sediments of the overland flow were enriched in C after the four rainfall events (Table 3). The results of the density fractionation clearly showed a large loss of C occluded in aggregates in the eroding zone, which was accompanied by an enrichment of C in the fPOC fraction in the other zones of the flume and in overland flow (Table 3). We assume that the disruption of macro-aggregates by raindrop peeling [35] and aggregate welding and development of a structural crust [17] resulted in the liberation of fPOC, which was preferentially transported [8]. The disruption of macro-aggregates will result in the release of micro-aggregates (smaller than 250 µm) from the macro-aggregates too. The C content of micro-aggregates within macro-aggregates is usually larger than that of macro-aggregates [36]–[38]. The release of such small aggregates and selective transport of small aggregates with low density [39] could be the reasons for the observed significant C enrichment of oPOC in sediments ranging from 2.3 to 3.2 (Table 3). However, we did not study aggregate stability and the detailed processes resulting in breakdown of the aggregates neither the related preferential erosion, transport and deposition of different sizes of aggregates and particles. That should be done in follow-up experiments.

The calculated mass balance of the experiment illustrates the disruption of aggregates in the eroding zone and the redistribution of C from aggregates to fPOC with an erosion-induced accumulation of fPOC in the sink area of 0.24 g C (Table 4). This accumulation is equal to an increase in fPOC by 48% comparing the source and the sink area. One logical source of this additional POC would be C occluded within aggregates in the eroding zone at the beginning of the experiment. This large accumulation of fPOC in the sink area contributed to the observed relative increase by 6% (Table 3) in the SOC content of the first two mm layer of the depositional zone.

**Table 4:** Soil organic C redistribution in three density fractions due to erosion (mass balance approach; C in free light fraction, fPOC; C in occluded light fraction, oPOC; C in heavy fraction, MOC).

Fraction	Source area (g C)^a^	Sink area (g C)			Relative value (% of SOC redistributed)			Erosion-induced fPOC^b^	Aggregate Breakdown oPOC^c^
		Depositional zone	Overland flow	CO_2_ emission	Depositional zone	Overland flow	CO_2_ emission	ΔC (g)	ΔC (g)
fPOC	0.5 (0.0)	0.6 (0.4)	0.14 (0.05)		4.5	1.0		+0.24	
oPOC	0.8 (0.0)	0.5 (0.1)	0.19 (0.04)		3.8	1.4			−0.11
MOC	12.3 (0.4)	9.8 (0.3)	2.13 (0.47)		72.1	15.7			
Total SOC/CO_2_	Σ 13.6	Σ 10.9 (0.6)	Σ 2.46 (0.60)	0.2 (0.0)	Σ 80.4	Σ 18.1	1.5		
SOC redistributed	Σ 13.6	Σ 13.6							

Results are given as mean and standard error of three replicates.

a . Original soil data were used to exclude any effect of soil erosion.

b . Erosion induced formation of fPOC (disruption of aggregates) ΔC (g) = fPOC in depositional zone + fPOC in overland flow- fPOC in source area.

c . Erosion-induced breakdown of aggregates (decline in oPOC) ΔC (g) = oPOC in depositional zone+oPOC in overland flow- oPOC in source area.

Particulate organic C already present in the soils and formed by disruption of aggregates (cf. above) was preferentially eroded and transported by overland flow as indicated by the largest CER ratio of the density fractions in any of the sampled soils and sediments. Per definition, fPOC is the lightest fraction, not associated with minerals and therefore easier to be translocated by water than soil particles with a higher density [23], [40], [41]. The high C enrichment of mineral-associated organic C (MOC) in the sediments of the overland flow suggested that water erosion separated the whole soil particles according to their density (Table 3). This fractionation occurred between the different density fractions. Increasing C concentrations (MOC<oPOC<fPOC; Table 3) resulted in increasing CER of the sediments in the same order.

A significant portion of the eroded and transported C enriched sediment was not retained in the downslope parts of the depositional zone and was exported by overland flow and left the flume (Table 4). Particularly the weakly decomposed C of the fPOC should be a readily available C and nutrient source for aquatic organisms [16], [42] contributing to CO_2_ emission from aquatic ecosystems. This process linking terrestrial and aquatic systems cannot be neglected for modeling the C cycle and has to be studied in more detail in future.

### Relationship between Erosion Rate and Carbon Enrichment

The inverse, non-linear relationship between the erosion rate and C enrichment of the sediments we found (Figure 3) is in agreement with previous studies [21], [40], [41], [43]. This inverse relationship is the result of increasing sediment concentration in the overland flow during each single event and from the first to the fourth rainfall event. One of the most important reasons for this relationship should be the breakdown of macro-aggregates by the raindrops as already discussed to be the main reason for the preferential erosion of fPOC [8], [10], [35]. This process should be particularly important at the beginning of each rainfall event because rewetting of dry soils results in the disruption of aggregates and the release of organic matter [44]. It is also reasonable to assume that the importance of this process will decrease with increasing number of rainfall events. Heavy rainfall causes compaction, welding and crust formation resulting in reduced infiltration and increased erosion and suspended solid concentration with time [15]. The preferential removal of C enriched soil will result in C enriched sediments particularly at the beginning of the experiment where the erosion rate was still small. After removal of this soil enriched in C, the erosion rate increases because of decreasing infiltration and generation of more overland flow. That will result in even increasing erosion rates because soils are less protected by organic matter and aggregation. In field situations, the relationship between erosion rate and C enrichment might be weaker because of continuous above and belowground C input and its positive effect on aggregation [45].

Decreasing C enrichment with large erosion rates, i.e. increasing sediment concentration, indicated that an increasing erosion rate does not result in proportionally increasing C losses. However, this does not mean that more severe erosion events lead to less impact on soil C. Very strong erosion events will translocate large amounts of C. However, this C might be better protected against further mineralization after deposition because C is mostly deposited as mineral associated C (Table 4). The C loading of mineral surfaces should be low as well, resulting in a more efficient stabilization against microbial decay [46], [47]. In addition, long-term erosion-induced C sequestration or depletion might depend on the precipitation frequency and intensity.

### Soil CO_2_ Effluxes

This study provides new data and insight on C decomposition under controlled conditions in an artificial landscape setting at eroding, transport and depositional positions allowing for a better process understanding. Although we did not scale up our results to the landscape level, it is important to know whether the fluxes measured do compare with observed field measurements and make any sense, also in comparison with previous indirect measurements on eroded sediments and soil profile investigations [5], [12]. In the present study, CO_2_ efflux rates measured (0.12 to 4.34 g C m^−2^ day^−1^) were in the range of soil respiration rates from agricultural loess soils [5], [19]. Initial increases of CO_2_ emissions immediately after each of our rainfall events might be explained by the increase in microbiological activity after re-wetting the dry soil and/or increased bioavailability due to aggregate breakdown [44]. Aggregate breakdown and subsequent exposure of previously encapsulated SOC provide substrates for microbial decomposition [8]. The re-wetting effect was particularly important after the first event and decreased during the course of the experiment. This is in line with a decreasing capacity of soil to release C from aggregates over time [48], [49].

Transport of topsoil and associated C influenced SOC decomposition rates at the different positions of the artificial slope. The small cumulative CO_2_ emission from the eroding zone should be the result of the observed preferential removal of C enriched materials (i.e. higher CER of the sediments at the depositional zone in comparison to the eroding zone, Table 3), which was either preferentially deposited or left the gutter. This preferential removal of more labile C (POC: fPOC and oPOC) left behind less C, which was relatively more stable (Table 3). In turn, accumulation of labile C fractions in the depositional zone contributed to an increasing difference in cumulative CO_2_ emission between the eroding and depositional zone over time (Figure 5).

The cumulative CO_2_ emission was significantly and positively related to fPOC (R^2^ = 0.94; *P* = 0.03) illustrating the more labile character of this SOC fraction. The large accumulation of fPOC probably explained that the transport zone had the largest cumulative CO_2_ emissions (Table 3 and Figure 7). Larger CO_2_ emissions from the depositional zone were anticipated because the deposited labile C (fPOC, oPOC) could be used as substrate and a source of energy for microbial respiration [50], [51]. Although CO_2_ emissions were large at the depositional zone, the SOC content increased by 6% in comparison to the control soils after four erosion events. Obviously, parts of the eroded and deposited SOC were preserved (Figure 5).

Considering all positions of the slope, mean DOC concentration in near-surface layers was positively correlated to median soil CO_2_ efflux rate (*P* = 0.02). The largest CO_2_ efflux was accompanied by largest DOC concentration in the transport zone – a second parameter (first fPOC) explaining the large CO_2_ efflux in this zone. Creed et al. [52] found that substrates (i.e. DOC) in the near-surface soil were strongly related to median soil CO_2_ efflux. Considering each position of the slope separately, median soil CO_2_ efflux rates were not significantly related to mean DOC concentrations at the eroding (*P* = 0.18), transport (*P* = 0.49) and depositional zones (*P* = 0.22). However, DOC was significantly correlated to the median soil CO_2_ efflux rate in the control soil (*P* = 0.05), which indicated DOC could be mineralized during the experimental period. Thus, DOC dynamics could not explain the observed additional C decomposition at the depositional zone. This might suggest a fast turnover of DOC or/and a direct use of POC by the microbial community.

### Total Carbon Budget

We estimated an erosion-induced C loss of 53 g C m^−2^ yr^−1^ calculated as the sum of net erosion-induced CO_2_ emission, C losses by overland flow (C in sediments, DOC) and through flow (cf. materials and methods, figure 7). During the entire experimental period, the averaged SOC fluxes leaving the flume with overland flow were 18 times larger than DOC fluxes including lateral fluxes by the through flow (Figure 7). Fluxes of DOC (0.26 g C m^−2^ yr^−1^) were rather low particularly due to decreasing DOC concentration during the experiment, i.e. with increasing number of events. Fluxes of sediment associated C were equivalent to 8.9% of the erosion-induced C loss while DOC fluxes were equivalent to 0.5% of those C losses. Therefore, sediment associated C played a much larger role than DOC in the erosion-induced linking of terrestrial and aquatic ecosystems. However, erosion-induced DOC flux should not be neglected because DOC might be particularly important for aquatic food webs [42], [53].

Erosion-induced CO_2_ emission was the dominant form of C loss, representing 90.5% of erosion-induced C loss. Based on the assumption made (cf. material and methods), 1.5% of total C redistributed (deposited C at the depositional zone plus C exported to aquatic ecosystems) was mineralized to CO_2_ (Table 4 and Figure 7). Previous estimates of decomposition of eroded SOC showed large variations, ranging from 0 to 100% (e.g. [7], [14], [15], [23]). In modelling studies the assumption is often used that at least 20% of the eroded SOC is decomposed as a consequence of soil erosion [7], [54]. Our measured values were much smaller than this conventional view of erosion effects on the C cycle. Palyakov and Lal [14] estimated 8% of SOC displaced by erosion had a potential to be mineralized and van Hemelryck et al. [23] estimated mineralization of 2% to 12% of the eroded SOC in a loess soils using laboratory rainfall simulation experiments. We propose three main reasons for the large difference. Firstly, the effects of the disruption of aggregates on extra CO_2_ efflux were relatively short-lived [48]. Secondly, C stabilization as affected by soil erosion and deposition might be underestimated in the previous assumption [5], [8]. Thirdly, the artificial slope was relatively short in our experimental setting in comparison to the field, which may result in an underestimation of transport effects on C mineralization.

## Conclusions

Rainfall simulation experiments are a useful approach to determine the role of soil erosion for the C cycle. The data of our 4-months experiment were comparable to field situations, despite of well-known shortcomings of laboratory approaches. The erosion rate was estimated to be 2.1 mm yr^−1^ (26 t ha^−1^ yr^−1^), which was comparable with estimations in this region based on field data [55]. Also C enrichment of exported sediments and soil CO_2_ efflux were in the range of field measurements.

Erosion-induced CO_2_ emission was the dominant form of C loss, representing about 90.5% of the erosion-induced C loss. In addition, a considerable amount of C rich sediments (265 g m^−2^ yr^−1^) was laterally exported by overland flow. Carbon associated with sediments was the main form of erosion-induced lateral C loss and not DOC. This exported C plays an important role in the connection of terrestrial and aquatic ecosystems.

In our experiment, this redistribution of C rich materials resulted in a net additional CO_2_ emission during transport and deposition. However, this enhanced CO_2_ emission is much smaller than previously thought. Most of the redistributed C by overland flow was bound to soil minerals (heavy fraction), which might be one reason for the unexpected small mineralization. As a consequence, the induced C sink by deposition could be larger than assumed.

Our study clearly demonstrated a fractionation of SOC upon erosion, transport and deposition controlling C mineralization. Disruption of macro-aggregates was identified as the main process responsible for the observed preferential redistribution of labile particulate organic C. Future studies should determine the conditions and processes resulting in breakdown of the aggregates and related preferential erosion, transport and deposition of different sizes of aggregates and particles. Furthermore, the replacement of carbon at eroding zones has to be included in future studies determining the role of soil erosion as a potential C source or sink.
